# Perillyl alcohol modulates activation, permeability and integrity of human brain endothelial cells induced by *Plasmodium falciparum*

**DOI:** 10.1590/0074-02760230033

**Published:** 2023-06-30

**Authors:** Adriana A Marin, Annette Juillard, Alejandro M Katzin, Leonardo JM Carvalho, Georges ER Grau

**Affiliations:** 1 Universidade de São Paulo, Instituto de Ciências Biomédicas, Departamento de Parasitologia, São Paulo, SP, Brasil; 2 University of Sydney, Department of Pathology, Vascular Immunology Unit, Sydney Medical School, New South Wales, Australia; 3 Fundação Oswaldo Cruz-Fiocruz, Instituto Oswaldo Cruz, Laboratório de Pesquisa em Malária, Rio de Janeiro, RJ, Brasil

**Keywords:** terpenes, human brain endothelial cells, P. falciparum, cerebral malaria, cell adhesion molecules

## Abstract

**BACKGROUND:**

Cerebral malaria (CM) is a severe immunovasculopathy caused for *Plasmodium falciparum* infection, which is characterised by the sequestration of parasitised red blood cells (pRBCs) in brain microvessels. Previous studies have shown that some terpenes, such as perillyl alcohol (POH), exhibit a marked efficacy in preventing cerebrovascular inflammation, breakdown of the brain-blood barrier (BBB) and brain leucocyte accumulation in experimental CM models.

**OBJECTIVE:**

To analyse the effects of POH on the endothelium using human brain endothelial cell (HBEC) monolayers co-cultured with pRBCs.

**METHODOLOGY:**

The loss of tight junction proteins (TJPs) and features of endothelial activation, such as ICAM-1 and VCAM-1 expression were evaluated by quantitative immunofluorescence. Microvesicle (MV) release by HBEC upon stimulation by *P. falciparum* was evaluated by flow cytometry. Finally, the capacity of POH to revert *P. falciparum*-induced HBEC monolayer permeability was examined by monitoring trans-endothelial electrical resistance (TEER).

**FINDINGS:**

POH significantly prevented pRBCs-induced endothelial adhesion molecule (ICAM-1, VCAM-1) upregulation and MV release by HBEC, improved their trans-endothelial resistance, and restored their distribution of TJPs such as VE-cadherin, Occludin, and JAM-A.

**CONCLUSIONS:**

POH is a potent monoterpene that is efficient in preventing *P. falciparum*-pRBCs-induced changes in HBEC, namely their activation, increased permeability and alterations of integrity, all parameters of relevance to CM pathogenesis.

Cerebral malaria (CM), one of the most serious infectious emergencies related to *Plasmodium falciparum* infection, mostly affects children aged 2-6 years in sub-Saharan Africa and young adults in South East Asia.^1^ Despite several available treatments, the lethality of CM still ranges from 15%-25%.^2^ This outcome is due to a neurovascular pathology characterised by the accumulation of both parasitised red blood cells (pRBCs) and host cells (leucocytes such as CD8^+^ T lymphocytes and platelets) in deep brain microvessels, leading to microcirculation impairment.^3^ ^,^ ^4^ ^,^ ^5^

Parasitised red blood cells sequestration in capillaries and post-capillary venules is mediated by their cytoadherence on endothelial cells through *P. falciparum* erythrocyte membrane protein 1 (PfEMP1), a step considered critical in CM pathogenesis.^6^ Several endothelial receptors, such as CD36, endothelial protein C receptor (EPCR), ICAM-1, E-selectin, thrombospondin, VCAM-1 and PECAM-1 are involved in the pRBCs cytoadherence to brain endothelium.^4^ ^,^ ^7^ ^,^ ^8^ This capillary and post-capillary venule obstruction severely reduces tissue blood flow, causing brain oedema and increasing intracranial pressure.^9^ If hypertension persists, tight junctions that are part of the endothelial barrier could collapse and lead to a breakdown of the brain-blood barrier (BBB). There is evidence that endothelial junctions are disrupted in patients with CM.^10^ Immunohistochemistry of post-mortem tissue from Vietnamese adults and Malawian children with CM showed a loss of endothelial cell (EC) junctional proteins ZO-1, occludin, and vinculin, most notably in vessels containing sequestered pRBCs.^10^ ^,^ ^11^ ^,^ ^12^ Therefore, the pathology caused by sequestration in CM is related to adhesion molecules and tight junction molecules in ECs.

On the other hand, there is evidence that extracellular vesicles (EV) released by the host, notably those derived from endothelial cells, play a pathogenic role in the severity of CM.^13^ ^,^ ^14^ Microvesicles (MVs), one type of EV, are also released upon *in vitro* cell stimulation and are a marker of cellular activation, apoptosis or tissue damage occurring *in vivo* in a variety of pathophysiological circumstances.^15^ ^,^ ^16^ ^,^ ^17^ For example, in CM patients, MVs released from platelets, ECs, monocytes and red blood cells (RBCs) are the most abundant and their levels significantly correlated with coma and thrombocytopenia.^18^ Also, studies in the murine model of CM (experimental cerebral malaria - ECM) demonstrated that MVs are not inert cellular products but active players in CM development. In fact, increased numbers of cell-specific MVs have been detected in ECM. Furthermore, adoptive transfer of MVs from mice with ECM to naïve mice caused breakdown of the BBB and pathology similar to ECM.^19^

To examine whether some interventions could prevent or revert *P. falciparum*-induced endothelial changes, we evaluated the effect of perillyl alcohol (POH) on human brain endothelial cells (HBEC) co-cultivated with pRBCs. Indeed, previous studies have shown that POH is a monoterpene highly effective to prevent cerebrovascular inflammation, breakdown of endothelial BBB and brain leucocyte accumulation in ECM models.^20^ ^,^ ^21^ In the present study, we evaluated if the loss of tight junction proteins (TJPs) expression in *P. falciparum*-induced HBEC can be improved by POH treatment. Therefore, we examined whether POH is able to downregulate endothelial activation, as assessed by ICAM-1 and VCAM-1 expression, and to reduce the excessive MV release by HBEC upon stimulation by *P. falciparum*. Finally, we studied the capacity of POH to revert HBEC monolayer permeability by monitoring trans-endothelial electrical resistance (TEER).

## MATERIALS AND METHODS

*HBEC and pRBCs cell culture* - Human brain endothelial cells (hCMEC/D3 cell line) were provided by Dr PO Couraud (Director, Institut Cochin, INSERM, Paris, France). The cells were grown in EBM-2 medium (Lonza CC-3156) until subconfluence in flasks coated with collagen 0.3% in a humidified atmosphere at 37ºC in 5% CO_2_. *P. falciparum* strains E8B were grown in RPMI+0.5% Albumax, as previously described.^22^ Late stage pRBCs were selected and concentrated using Voluven 6% to reach an average of 80-90% pRBCs.

*Co-culture of HBEC and pRBCs* - HBEC were seeded on coverslips coated with collagen 3% into a 24 well plate until they reached subconfluence. Then, tumour necrosis factor (TNF) activation of HBEC was carried out by treating the cells with 10 ng/ml TNF for 24 h. pRBCs were then co-cultivated with HBEC at a 50 pRBCs: 1 HBEC ratio for 48 h. A stock solution of POH (Santa Cruz Biotechnology (CAS 57717-97-2) was prepared in ethanol and then used to prepare a 20 μM (final POH concentration) solution in RPMI or EBM-2 medium (0.5% ethanol final concentration), which was added simultaneously, 6 or 12 h after of pRBCs overnight. Uninfected red blood cells in RPMI or EBM-2 medium with 0.5% ethanol were used as negative control. Finally, the supernatant was removed, and each well was washed carefully three times during 20 min with EBM2 pre-warmed medium.

*Immunofluorescence assay* - 7’000 HBEC were seeded on coated glass coverslips previously treated with 3% type I collagen solution. pRBCs were then co-cultivated with HBEC at a 50 pRBCs: 1 HBEC ratio for 48 h. 20 μM of POH [Santa Cruz Biotechnology (CAS 57717-97-2)] was added simultaneously, 6 or 12 h after of pRBCs overnight. They were then fixed after 48 h using 4% paraformaldehyde diluted in bovine serum albumin (BSA) for 30 min. All cells were washed and blocked with 5% BSA for 1 h. Cells were incubated overnight at 4ºC with primary antibodies diluted in 1% BSA [Anti-JAM-A (ab180821) 1/200; Anti-occludin (ab216327) 1/100; Anti-VE-cadherin (348502) 1/50; Anti-ICAM-1 (ab109361) 1/500; Anti-VCAM-1 (ab134047) 1/100]. Cells were then washed three times with phosphate-buffered saline (PBS) and incubated with secondary antibodies for 1 h at room temperature (Alexa Fluor 488 donkey anti-mouse IgG and Alexa Fluor 488 goat anti-rabbit IgG). Cells were washed three times for 10 min with PBS. DNA was stained with 4′,6-diamidino-2-phenylindole dihydrochloride (DAPI, Sigma-Aldrich) for 5 min. Cells were washed another three times with PBS to remove all free DAPI. The coverslips were placed on 3 μL Aqueous Mounting Media on glass slides. To quantify VE-cadherin (VE-cad), occludin-1, JAM-A, ICAM-1 and VCAM-1 levels on HBEC, at least 10 photos per slide were performed in different areas using an Olympus BX51 microscope. These micrographs were then converted to binary images using ImageJ (v1.48, NIH) for analysis. Area fraction (surface occupied by selected cell), mean fluorescence, and integrated density of fluorescence were measured. The total corrected cellular fluorescence (TCCF) = Integrated density- (area of selected cell × mean fluorescence) was calculated.^23^ Data normalisation was made using the TCFF against the number of cells (using DAPI) per field.

*Trans-endothelial electrical resistance measurement* - TEER was monitored during 72 h using an electric cell-substrate impedance sensing (ECIS) system (Applied BioPhysics). Wells were coated with 200 μL of 3% rat type I collagen (Sigma) for 30 min and pre-treated with 200 μL of L-cysteine during 15 min at room temperature. HBEC were seeded at 25,000 cells/wells in 8-well slides and allowed to grow for two to three days, until confluence, in complete HBEC medium. Confluent HBEC monolayers (estimated 150,000 cells/well) were activated with TNF (10 ng/mL) for 24 h, as previous experiments have shown that this does not modify TEER by itself. pRBC were added directly to wells at a ratio of 50 pRBC/HBEC and then 20 µM of POH was added. Histamine (100 mM), which causes a rapid decrease in TEER, was used as a positive control. POH alone on unstimulated cells was used as the control.

*Quantification of MVs by flow cytometry* - After co-culture of HBEC and pRBC for 24 h, the supernatant was collected and centrifuged during 10 min at 1,800 g to remove debris. A second centrifugation for 45 min at 18,000 g was made to pellet MV. The pellet was collected and kept at -80ºC until annexin V-FITC/7-AAD kit (Beckman Coulter) analysis. Briefly, 20 µL of sample was mixed with 2 µL of binding buffer and 1 µL Annexin and incubated 20 min at room temperature in the dark. Then, were diluted 10x (1:10) binding buffer with water and added 180 µL in each well. 200 µL were taken and placed in a flow tube for reading. Acquisition and analysis of data were performed using a Gallios MPL cytometer and the Kaluza software (Beckman-Coulter), respectively.

*Statistical analysis* - For comparison of means of more than two treatments, variables with normal distribution were analysed using one-way analysis of variance, and other variables were analysed using non-parametric Kruskal-Wallis tests, with Tukey’s or Dunn’s post-test. For comparison of means between two groups, a Mann-Whitney-test was used. The analyses were performed using GraphPad PRISM^®^ software version 5.3. Values of p < 0.05 were considered statistically significant.

## RESULTS

**Fig. 1:**
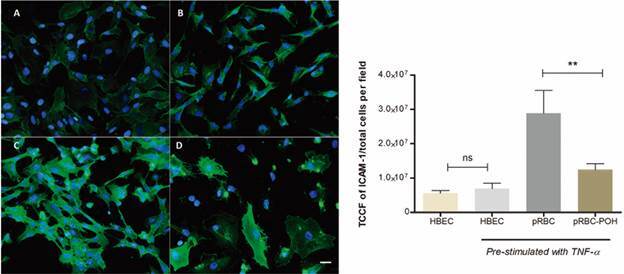
perillyl alcohol (POH) downmodulates intercellular cell adhesion molecule 1 (ICAM-1) upregulation in human brain endothelial cells (HBEC) caused by *Plasmodium falciparum*. (A) Unstimulated HBEC showed basal expression of ICAM-1 (green), and nucleus with 4′,6-diamidino-2-phenylindole dihydrochloride (DAPI) (blue). (B) HBEC pre-stimulated with tumour necrosis factor (TNF) for 24 h. (C) HBEC co-cultivated with parasitised red blood cells (pRBCs) during 24 h. (D) HBEC co-cultivated with pRBCs and treated with POH for 24 h. The data are represented as mean ± standard deviation (SD). TCCF: total corrected cellular fluorescence. This graph is representative of three different experiments. **p < 0.01. Scale bar: 50 µm.

**Fig. 2:**
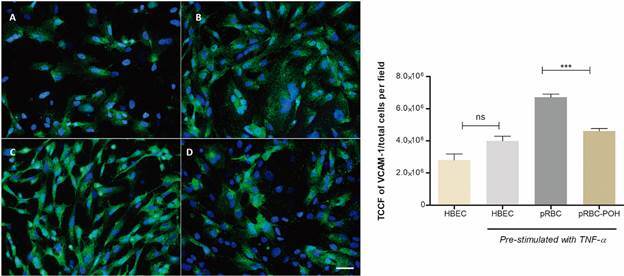
perillyl alcohol (POH) downmodulates vascular cell adhesion molecule 1 (VCAM-1) upregulation in human brain endothelial cells (HBEC) caused by *Plasmodium falciparum*. (A) Unstimulated HBEC showed basal expression of VCAM-1 (green), and nucleus with 4′,6-diamidino-2-phenylindole dihydrochloride (DAPI) (blue). (B) HBEC pre-stimulated with tumour necrosis factor (TNF) for 24 h. (C) HBEC co-cultivated with parasitised red blood cells (pRBCs) during 24 h. (D) HBEC co-cultivated with pRBCs and treated with POH for 24 h. The data are represented as mean ± standard deviation (SD). TCCF: total corrected cellular fluorescence. This graph is representative of three different experiments. ***p < 0.001. Scale bar: 50 µm.

*Perillyl alcohol reduces the ICAM-1 and VCAM-1 upregulation on P. falciparum-stimulated HBEC* - There is substantial evidence that CM involves an upregulation of cerebral endothelial adhesion molecules that mediate the cytoadherence of pRBCs to blood vessels. We thus evaluated if POH treatment could downregulate ICAM-1 and VCAM-1 expression by HBEC. Figs 1 and 2 show that POH treatment indeed downregulated the increase of both ICAM-1 and VCAM-1 caused by pRBC. Unstimulated HBEC showed a basal expression of ICAM-1 and VCAM-1. HBEC prestimulated with TNF for 24 h had an increase of both VCAM-1 and ICAM-1 expression. When co-cultivated in the presence of pRBCs for 24 h, HBEC, as expected, upregulated both VCAM-1 and ICAM-1. In HBEC treated concomitantly with pRBC and POH, ICAM-1 and VCAM-1 expressions were significantly downregulated (p < 0.05).

**Fig. 3:**
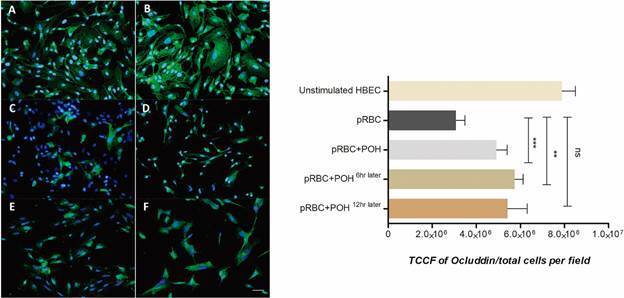
perillyl alcohol (POH) modulates occludin-1 expression on human brain endothelial cells (HBEC). (A-B) HBEC unstimulated showed a normal ocluddin-1 expression (green) and nucleus with 4′,6-diamidino-2-phenylindole dihydrochloride (DAPI) (blue). (C) HBEC co-cultivated with parasitised red blood cells (pRBCs). (D) HBEC co-cultivated with pRBC and co-treated with 20 µM POH (E-F) HBEC co-cultivated with pRBC and treated with 20 µM of POH, 6 and 12 h after pRBC, respectively. Data are expressed as mean ± standard deviation (SD). TCCF: total corrected cellular fluorescence. This graph is representative of three different experiments. ***p < 0.001; **p < 0.01; NS p >0.05. Scale bar: 40 µm.

**Fig. 4:**
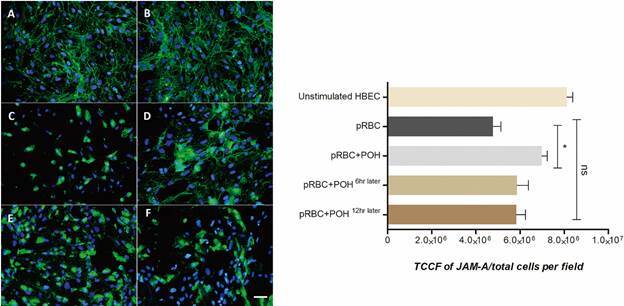
perillyl alcohol (POH) modulates junctional adhesion molecule A (JAM-A) expression on human brain endothelial cells (HBEC). (A-B) unstimulated HBEC showed a normal JAM-A expression (green) and nucleus with 4′,6-diamidino-2-phenylindole dihydrochloride (DAPI) (blue). (C) HBEC co-cultivated with parasitised red blood cells (pRBCs). (D) HBEC co-cultivated with pRBC and co-treated with 20 µM POH. (E-F) HBEC co-cultivated with pRBC and treated 20 µM of POH, 6 and 12 h after pRBC, respectively. The data are expressed as mean ± standard deviation (SD). TCCF: total corrected cellular fluorescence. This graph is representative of three different experiments. *p < 0.05; NS p > 0.05. Scale bar: 40 µm.

**Fig. 5:**
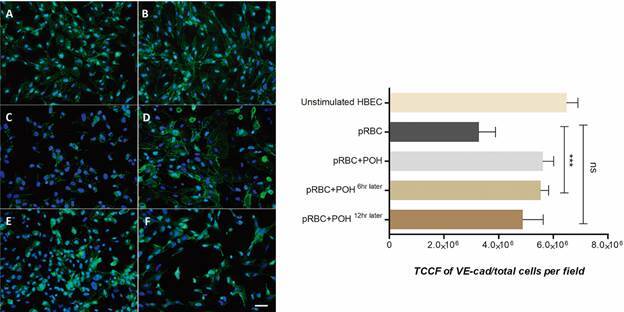
perillyl alcohol (POH) prevents vascular endothelial (VE)-cadherin modulation on human brain endothelial cells (HBEC). (A-B) unstimulated HBEC showed a normal VE-cadherin expression (green) and nucleus with 4′,6-diamidino-2-phenylindole dihydrochloride (DAPI) (blue). (C) HBEC co-cultivated with parasitised red blood cells (pRBCs). (D) HBEC co-cultivated with pRBCs and co-treated with 20 µM POH (E-F) HBEC co-cultivated with pRBCs and treated 20 µM of POH, 6 and 12 h after pRBCs, respectively. The data are expressed as mean ± standard deviation (SD). TCCF: total corrected cellular fluorescence. This graph is representative of three different experiments. ***p < 0.001; NS p > 0.05. Scale bar: 40 µm.

*Perillyl alcohol modulates the P. falciparum effect on TJPs expression on HBEC* - To investigate the effect to POH on the *P. falciparum*-induced alteration of HBEC integrity, TJP expression was quantified. HBEC were prestimulated with TNF was performed in order to increase pRBC adherence and then co-cultivated them with *P. falciparum* at a 1:50 ratio for 48 h. Figs 3 - 5 show the occludin-1, JAM-A and VE-cadherin expression in HBEC with different treatments, respectively. The results showed a significant reduction of occludin-1, JAM-A and VE-cad expression in HBEC co-cultivated with pRBCs compared with the control cells. Interestingly, in HBEC treated concomitantly with pRBCs and POH, the expression of TJP was significantly less decreased compared to levels seen with pRBC alone (p < 0.001). To evaluate whether POH could modulate pre-existing, pRBC-induced TJP decreased expression on HBEC, POH treatment was added 6 h after pRBC co-culture. Indeed, POH was able to revert the pRBC effect on monolayer integrity as well (p < 0.01). However, POH treatment added 12 h later did not have any effect on TJP expression.

**Fig. 6:**
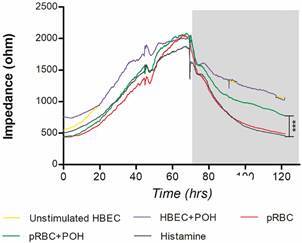
perillyl alcohol (POH) increases trans-endothelial electrical resistance (TEER) of *Plasmodium falciparum* induced human brain endothelial cells (HBEC) TEER was monitored during 72 h using electric cell-substrate impedance sensing (ECIS). HBEC were seeded in 8-well slides and allowed to grow for two to three days, until confluence. Confluent HBEC monolayers were activated with tumour necrosis factor (TNF) (10 ng/mL) for 24 h parasitised red blood cells (pRBCs) were added directly at a ratio of 50 pRBCs/cell and 20 µM of POH was added concomitantly. Histamine (100 mM) was used as a positive control. POH alone on unstimulated cells was used as the control. The grey shaded area represents the period during which pRBCs and other compounds were added to HBEC monolayers. Significant differences relative to pRBC-treated HBEC; ***p < 0.001.

*Perillyl alcohol increases TEER of HBEC co-cultured with P. falciparum pRBCs* - ECIS was used to assess the TEER of the HBEC monolayer, which reflects changes in the permeability of the endothelial monolayer. HBEC co-cultured with pRBCs decreased significantly their TEER compared with unstimulated HBEC. After 3 h of incubation with pRBC, TEER values were significantly decreased when compared with those from unstimulated HBEC. Remarkably, addition of POH simultaneously with pRBCs prevented this decrease of TEER. TEER of HBEC co-cultured with pRBCs and co-treated with POH was significantly higher compared with HBEC co-cultured with pRBCs and remained constant during 48 h. Unstimulated cells with POH treatment alone showed no changes in cell membrane dynamics (Fig. 6).

**Fig. 7:**
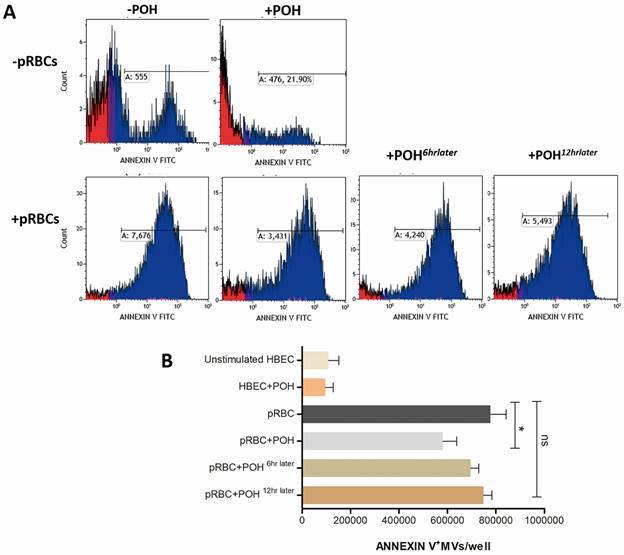
perillyl alcohol (POH) reduces the microvesicle (MV) release by *Plasmodium falciparum-*induced human brain endothelial cells (HBEC). After HBEC and parasitised red blood cells (pRBCs) were co-cultured for 24 h, the supernatant was collected from each well and processed for multiple centrifugations. Pellet was collected and kept at -80ºC until Annexin V-FITC/7-AAD kit (Beckman Coulter) analysis. (A) Histograms of Annexin-V^+^ events from HBEC co-cultivated with pRBC and treated with POH, in the indicated conditions. (B) Quantification of Annexin-V+ events from HBEC treated as in A. Data are represented as mean ± standard deviation (SD). POH alone on unstimulated cells was used as the control. This graph is a combination of five different co-culture experiments. *p < 0.05; NS p > 0,05.

*Perillyl alcohol prevents MV release from P. falciparum-stimulated HBEC* - In order to explore POH effects on the release of MVs by HBEC in the presence of pRBC, we quantified the release of MVs in these conditions. The addition of pRBC to HBEC increased the numbers of MVs in the culture supernatant more than seven times compared with the MVs release from unstimulated HBEC. POH treatment was able to significantly prevent this increased MVs release from HBEC caused by pRBC (p < 0.05). However, POH late treatment was not able to significantly reduce MV release, demonstrating that POH can prevent but not revert the HBEC production of MVs induced by *P. falciparum.* Unstimulated cells with POH treatment alone showed no changes in cell membrane dynamics (Fig. 7).

## DISCUSSION

Perillyl alcohol is a monoterpene that has been previously shown to significantly prevent mortality in an experimental model of cerebral malaria, and this effect was associated with prevention of blood brain barrier breakdown and cell adhesion expression in *Plasmodium berghei* ANKA (PbA)-infected mouse brain, along with reduced systemic inflammation.^20^ ^,^ ^21^ BBB breakdown is a feature of both CM^24^ and ECM,^25^ causing an increase in vascular permeability and allowing the entry of inflammatory mediators into the brain.^26^ In the present study, addition of POH to brain endothelial cells co-cultivated with *P. falciparum* pRBCs showed high efficacy in downregulating endothelial activation and permeability changes induced by the parasite. Indeed, as expected, *P. falciparum* pRBCs induced endothelial activation, with increased expression levels of ICAM-1 and VCAM-1, loss of barrier integrity with decreased levels of occluding, JAM-1 and V-cadherin, as well as a decrease in TEER in the endothelial monolayer. All these aspects of endothelial activation and dysfunction were prevented by the addition of POH to the culture, corroborating the *in vivo* findings in PbA-infected mice.

*Plasmodium falciparum* pRBCs are known to adhere to endothelial cells through the binding of PfEMP-1 variants to diverse endothelial cell receptors, and PfEMP-1 variants leading to parasite sequestration in the brain bind to receptors such as EPCR, ICAM-1, and VCAM-1.^4^ ^,^ ^7^ ^,^ ^8^ ^,^ ^27^ Systemic inflammation, with increased levels of cytokines such as TNF, increases the expression of these receptors, facilitating parasite adhesion and accumulation. POH has been previously shown to decrease systemic inflammatory cytokine levels and splenic lymphocyte activation and proliferation in PbA-infected mice.^20^ ^,^ ^21^ Therefore, the observed decrease in cell adhesion molecule expression in the brain of the POH-treated animals could be interpreted as an effect secondary to the prevention of splenic lymphocyte activation^28^ leading to systemic inflammation. However, the present *in vitro* data indicate that POH has a direct anti-inflammatory action on endothelial cells, causing substantive reductions in ICAM-1 and VCAM-1 expression in TNF- and pRBC-stimulated brain endothelial cells. The mechanism behind this direct effect on the endothelial cells is unknown and deserves further investigation. A key element in brain endothelial cell activation induced by proinflammatory stimuli is increased blood-brain barrier permeability consequent to loss of tight-junction integrity.

Activation of the endothelial cells has been linked to sequestration of pRBCs and TNF-overproducing monocytes.^29^ ^,^ ^30^ ^,^ ^31^ ^,^ ^32^ ^,^ ^33^ ^,^ ^34^ Damage to the BBB allows leakage of plasma proteins and fluids into the perivascular and parenchymal extracellular spaces, causing vasogenic oedema and endothelial activation.^35^ Previous studies have demonstrated a decrease in TJPs in both CM and ECM,^36^ and these events were reproduced *in vitro* in the present study, with brain endothelial cell monolayers showing marked decreases in the expression levels of occludin, JAM-1 and VE-cadherin when co-cultivated with *P. falciparum* pRBCs. Addition of POH at the beginning of co-culture significantly prevented the loss of these tight-junction proteins, again indicating substantive protection of endothelial cell barrier integrity. Addition of POH even 6 hours, but not 12 h, after endothelial cells and pRBC co-cultivation still resulted in improved TJPs. These findings indicate that POH can not only prevent, but also help reverse, tight junction damage when timely administered. This effect is of critical importance, as increased BBB permeability persists and even continues to deteriorate in the first hours following artemether treatment in mice with ECM.^37^ ^,^ ^38^

Endothelial cell damage leading to vascular dysfunction and BBB breakdown in cerebral malaria results from multifactorial aggression involving pRBC binding and sequestration and a potent inflammatory response.^39^ More recently, an important role for MVs derived from various sources such as endothelial cells, monocytes, platelets, red blood cells and the parasite has been established in human and experimental cerebral malaria.^14^ In CM patients, MV abundance has been correlated with the depth of coma and degree of thrombocytopenia^18^ Cytokine stimulated brain endothelial cell MVs can interact with T cells in an ICAM-1- and VCAM-1-dependent fashion^14^ and infected erythrocyte MVs can carry functional miRNA that can be internalised by endothelial cells.^14^ MVs have also been shown to be uptaken by astrocytes.^40^ All these observations indicate that MVs from different sources may play key roles in modulating endothelial cell and BBB functions in cerebral malaria. In the present study, a 7-fold increase in the release of MVs was observed when HBEC were incubated with *P. falciparum* pRBCs. Addition of POH at the beginning of the HBEC-pRBCs co-culture resulted in a partial (~ 25%) but significant inhibition of MV release by HBEC. No effect was observed when POH was added 6 or 12 h after the beginning of co-culture. Together with the data on cell adhesion molecule and tight junction molecule expression and also on TEER, these findings indicate that POH is not able to completely prevent endothelial cell activation, but the partial effect seems to be sufficient for brain endothelial cells to maintain barrier integrity.

The *in vitro* findings using co-cultures of HBEC and *P. falciparum* pRBCs in this study add to the findings in the ECM model^20^ ^,^ ^21^ as they indicate direct effects of the compound on endothelial cells, thereby substantiating POH as a promising candidate for further studies. Understanding the molecular targets of POH action could provide valuable insights into CM pathogenesis and guide future effective interventions aimed at preventing or reversing endothelial cell damage and BBB breakdown in CM.
